# Supplementary material for: Are gamers better laparoscopic surgeons? Impact of gaming skills on laparoscopic performance in “Generation Y” students
Source: PLoS One. 2020 Aug 26;15(8):e0232341. doi: 10.1371/journal.pone.0232341 (PMC7449406; doi:10.1371/journal.pone.0232341)
Supplement: Attachment — Submitted filename: Peer Review PONE-D-19-23296.pdf [file pone.0232341.s001.pdf]

Review of “Are Gamers better laparoscopic surgeons? Impact of Gaming Skills on Laparoscopic Performance in “Generation Y” students”

Manuscript Number: PONE-D-19-23296
Reviewed by: Yin Jun Hao
Email: mpeyjh@nus.edu.sg

Summary

The study discussed a possible connection between laparoscopic surgery capabilities and the skill of computer games. The study used two custom made games (one 2D and one 3D spaceship centred game) and three laparoscopic tasks (rope pass, paper cut, and peg transfer) to measure an individual’s gaming and laparoscopic surgery capabilities respectively. At the end, the study managed to obtain a finding of a small significant correlation between the performance of the laparoscopic task and the games.

Discussion

The efforts for this study are commendable as the creation of the custom games are not easy to create and additional work of inviting the professional Esports players to prove that the custom games are not easy for non-experienced players. Nonetheless there are certain areas of discussion and improvement which will benefit this study in the long run.

Factors surrounding the 2 methods of comparisons

The types of games played in the Esports industry in the recent era consists heavily of Multiplayer Online Battle Arena games (e.g. Dota 2), Battle Royale games (e.g.

PUBG) and First Person Shooting games (e.g. Overwatch). These games require one to have good action per minute capabilities and critical thinking within a short period of time. And this coincides with the needed skills to perform well for the custom games and players are given stressful conditions and the need to react to the obstacles which increases with speed throughout the games. Which are shown through the professional Esports players performing better in the custom games as compared to non-professionals. However, the skills required for laparoscopic tasks are mainly precision and visuospatial cognition. Thus, the custom games created may not be the best for this study. One suggestion could be changing the conditions of the custom game. For example, instead of making the game progressively harder through speed, the obstacles could instead get larger, making the area of allowable mistake smaller when passing though the obstacles.

Purpose and aim of the study

The purpose of the study was not coherent throughout the paper. It was discussed that the possibility of the connection between laparoscopic surgery and computer games could bring about a change in recruitment and training (background, line 28-29) but towards the end of the paper, it was mentioned that using games to train laparoscopic skills may not be efficient (discussion, line 417-426). In addition, it is not fair to judge a person's potential of laparoscopic skill through the use of video games as both of these skills require different skill sets. Although the study did manage to find a possible correlation between laparoscopic surgery and computer games capabilities, it will be more purposeful if there is a coherent purpose. One suggestion could be finding out that through the use of video games, one is able to improve the

critical thinking of the individual due to the short amount of time given to react to events in the game. This finding could result to a possible change to how laparoscopic simulations are conducted. For example, having time limits and creating a stressful environment as used for the custom games (Definition of the requirements for custom video games, line 112-113).

Adding in an additional pool of subjects

One interesting suggestion is to have the professional Esports players participate in the laparoscopic task. The findings might give more insights for this study as the professional Esports players are definitely considered as experienced players for the comparison of the study. And this could further substantiate the intended aim of the study which is to find the connection between the level of both the potential laparoscopic surgery abilities and gaming skill.
